# CBCT Analysis of Dento-Skeletal Changes after Rapid versus Slow Maxillary Expansion on Deciduous Teeth: A Randomized Clinical Trial

**DOI:** 10.3390/jcm11164887

**Published:** 2022-08-20

**Authors:** Marco Serafin, Rosamaria Fastuca, Alberto Caprioglio

**Affiliations:** 1 Department of Biomedical Sciences for Health, University of Milan, 20133 Milan, Italy; 2 Independent Researcher, 21100 Varese, Italy; 3 Department of Biomedical, Surgical and Dental Sciences, Section of Orthodontics, University of Milan, 20122 Milan, Italy; 4 Fondazione IRCCS Cà Granda, Ospedale Maggiore Policlinico, 20122 Milan, Italy

**Keywords:** CBCT, leaf expander, rapid maxillary expansion, randomized controlled trial, slow maxillary expansion

## Abstract

The aim of the present study was to compare skeletal and dental changes after rapid maxillary expansion (RME) and slow maxillary expansion (SME) performed by a Leaf Expander (LE) with upper deciduous teeth as anchorage and using 3D CBCT (Cone Beam Computed Tomography) analysis. Mixed dentition patients were randomly divided in two groups, according to the different expansion used anchored on maxillary primary second molars: the RME group (*n* = 16) was treated with a Hyrax type expander, whereas the SME group (*n* = 16) was treated with an LE expander. CBCT scans were performed before (T1) and after treatment (T2) and analyzed with a custom landmarks system. A paired *t*-test was used for intragroup analysis between T1 and T2, and a Student t-test was used for intergroup analysis; statistical significance was set at 0.05. Both RME and SME groups showed a statistically significant increase in dental and skeletal diameters. Group comparisons between T1 and T2 showed a significant expansion rate in the RME group for upper permanent molars (*p* = 0.025) but not for deciduous molars (*p* = 0.790). Moreover, RME showed higher increases for skeletal expansion evaluated at nasal walls (*p* = 0.041), whereas at pterygoid plates did not show any significant differences compared with the SME group (*p* = 0.849). A significant transverse expansion could be achieved with the expander anchored on deciduous teeth. RME and SME produced effective both skeletal and dentoalveolar transverse expansion; RME produced more anterior expansion than SME but less control regarding the permanent molar decompensation. SME by LE therefore could be an efficient and helpful alternative in the treatment of transverse maxillary deficiency in growing patients.

## 1. Introduction

Maxillary expansion is an efficient procedure to correct maxillary transverse discrepancy. There are different modalities of expansion and among them rapid protocols are characterized by heavy intermittent forces applied in short lapses of time, meanwhile slow protocols use continuous lower forces applied over long periods.

Rapid Maxillary Expansion (RME) increases molar and canine diameters [1] and enlarges the upper arch through orthodontic and orthopedic effects [2]. RME can also be useful in class II malocclusion characterized by mandibular retroposition caused by maxillary transverse discrepancy not immediately evident due to occlusion of maxillary posterior teeth on narrower sectors of the mandible [3]. Expansion rate is a helpful parameter to study maxillary modifications. Rapid activation is usually performed at a rate of two turns per day providing 0.25 mm per turns. To improve stability, overexpansion and at least 6 months of retention are required [4]. Studies on cellular midpalatal suture have shown an immediate sutural density reduction after RME and an increase after 6 months, indicating the need for adequate retention time for suture reorganization [5]. RME affects not only midpalatal suture, but also circumaxillary sutures [6]. Although it is a treatment commonly used by clinicians, it might present some disadvantages: when the appliance is anchored to permanent teeth, it might cause their buccal tipping, root resorption, bone fenestration, and reduced buccal bone thickness [7,8]. To avoid these effects, palatal expansion in growing patients can be achieved by using primary teeth as anchorage [2,9]. It ensures a more stable anterior expansion and a self-expansion of permanent molars [10].

An efficient alternative to RME is slow maxillary expansion (SME), which occurs at a low-force rate that allows tissues physiological adaptation. SME generates less tissue resistance of circumaxillary structures and a physiological maxillary reorganization, reducing the accumulation of RME residual orthopedic forces and minimizing post expansion relapse [11]. SME can be achieved with the same appliance as RME with different activation protocols, or with different appliances. Leaf Expander (LE) [12] was recently suggested as an SME expander characterized by the application of light and constant forces with predetermined intensity. It presents a double nickel titanium leaf spring that recovers its original shape during deactivation, resulting in a calibrated maxillary expansion. LE is typically anchored on deciduous teeth, avoiding undesirable effects on permanent teeth.

However, it is important to first clinically diagnose the presence of any transversal skeletal discrepancy between the maxilla and the mandible. With upper and lower posterior teeth optimally positioned by eliminating dental compensation within their respective alveolar bone processes, comparison between the distances from cusp-to-cusp (maxillary arch) and fossa-to-fossa (mandibular arch) can accurately diagnose the presence of transversal hypoplasia and the need for maxillary orthopedic expansion [13].

To date, in absence of scientific evidence on which is the best expansion protocol with appliance anchored on primary teeth, the choice of the type of expansion depends just on practitioner’s experience. The aim of this study was to evaluate skeletal and dental changes in growing patients after RME and SME with appliance anchored on upper deciduous teeth by 3D Cone Beam Computed Tomography (CBCT) analysis. The null hypothesis was that no differences were detected between the two protocols about the amount of dentoskeletal expansion, in particular regard to upper first permanent molars and basal maxillary bone.

## 2. Materials and Methods

### 2.1. Population and Study Design

The protocol for this randomized clinical trial was reviewed and approved by the Ethical Committee of the University of Milan (protocol number ROS18/02) and the following procedures adhered to the World Medical Organization Declaration of Helsinki. The sample was selected according to the following inclusion criteria: (I) early mixed dentition; (II) first permanent molars erupted and non-mobile maxillary deciduous second molars in accordance with the radiographic method purposed by Primozic et al., [14]; (III) skeletal class I or II; (IV) maxillary transverse discrepancy with or without posterior crossbite evaluated by CBCT measurements of transversal dimension of basal bone at the level of upper and lower first permanent molars. Exclusion criteria were: (I) craniofacial anomalies; (II) noticeable asymmetries; (III) previous orthodontic treatment; (IV) skeletal class III.

All the subjects presenting at the Department of Orthodontics of University of Milan meeting the inclusion and exclusion criteria were enrolled in the study between March 2020–January 2022. Informed consent for scientific purposes was obtained priorly to start any treatment.

### 2.2. Sample Size Calculation

Sample size was calculated considering as primary outcome the amount of expansion at the upper permanent molars. A clinically relevant difference between means of 1.91 mm with a combined standard deviation (SD) of 1.75 mm derived from a pilot study performed on 3 patients per group was established. To retrieve β = 0.80 with α set at 0.05 a sample of at least 14 patients per group was needed. A 10% dropout of patients should be considered, then a greater number of patients was enrolled for a total number of 18 patients per group.

### 2.3. Randomization Procedure

**Figure 1:**
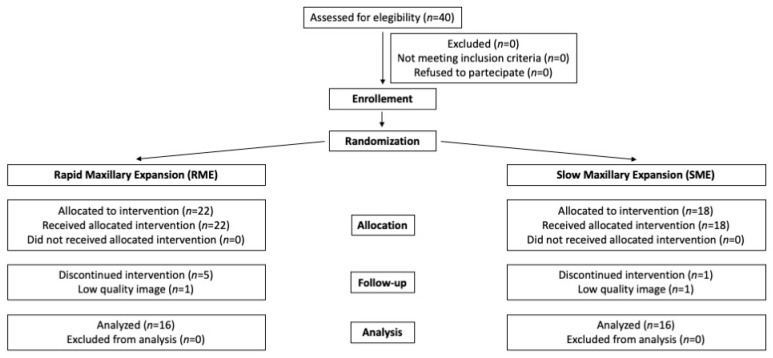
Consort flow diagram.

Every new patient adherent to inclusion and exclusion criteria was allocated to the RME group or the SME group using a computer program (www.random.org, accessed in 22 March 2022). Then, randomization results were consulted by statistician every time a new patient was enrolled. Patients who did not follow the activation protocol, did not come back for appointments, or whose structures were difficult to visualize on CBCT, were dropped from the study sample. The study was blinded regarding the statistical analysis, eliminating from the elaboration file every reference to patient group assignment. A consort diagram showing the flow of patients through the trial is provided in Figure 1. Finally, the RME group comprised 16 patients (12F, 6M) with a mean age of 8.50 ± 1.39 years and the SME group comprised 16 patients (11M, 5F) with a mean age of 8.96 ± 0.92 years.

### 2.4. Intervention and Outcomes

Each patient underwent two CBCT scans prior to the beginning of expansion (T1) and immediately after the removal of expander (T2), an average of 9 months after the treatment start with no other orthodontic treatment; DICOM files were processed in Mimics Software (Materialise NV, Leuven, Belgium). For each CBCT image, skeletal and dental landmarks (Table 1) were taken using a coordinate system proposed by Lagravère et al., and confirmed in a similar study by Serafin et al., [2,15] and measurements (Table 2) performed.

The RME group was submitted to a Hyrax expander anchored to the maxillary deciduous second molars by bands and with palatal pads bonded on primary cuspids. The screw (11 mm long) of the palatal expander (A0620-11; Leone, Firenze, Italy) was initially turned twice (0.50 mm). Afterwards, parents of patients were instructed to turn the screw once per each following day (0.25 mm per day). The expander remained passive in situ over the following 7 months.

The SME group was submitted to an LE (A2703-06, Leone, Firenze, Italy) anchored to the maxillary deciduous second molars by bands and cuspid’s palatal rests; an expander’s screw of 6mm long and 450 g force was used. The first 3 mm of expansion happened spontaneously, when ligature was removed after bonding appliance. Afterwards, the LE was reactivated by recompressing the Ni-Ti spring for 3 times (1 mm per month). The expander was kept passive in situ over the following 6 months.

Maxillary expansion was performed until dental overcorrection for each patient from both RME and SME groups. The primary outcomes measured for this trial were skeletal and dental changes after RME and SME with LE on upper deciduous teeth. All the intervention and measurements were performed by the same trained operator (M.S.) and checked by expert operators (R.F. and A.C.) in case of doubt.

### 2.5. Statistical Analysis

SPSS software (v.22.0; IBM Corp., Armonk, NY, USA) was employed to perform statistical analysis. The Shapiro–Wilk test revealed a normal distribution of data and, therefore, parametric tests were employed for analysis. A Student t-test was used to compare groups at starting forms. A paired t-test was used to compare each variable within the same group between T1 and T2 and a Student *t*-test was used to compare each variable between the two groups with a 5% significance.

## 3. Results

Mean and standard deviations were calculated for all variables for both timepoints in both groups.

Starting forms comparisons (Table 3) did not show significant differences between groups, except for upper permanent molars buccolingual inclination (FURCA16_PC16_PC26, *p* = 0.019; FURCA26_PC26_PC16, *p* = 0.043), significantly higher in the SME group at the beginning of treatment.

The RME group showed statistically significant increases in most of the tested variables between T1 and T2 (Table 4), except for nasal floor expansion (RNF_LNF, *p* = 0.157) and upper molars buccolingual inclination (FURCA16_PC16_PC26, *p* = 0.647; FURCA26_PC26_PC16, *p* = 0.913).

The SME group showed statistically significant differences between T1 and T2 in various measurements (Table 5), exhibiting permanent and deciduous molars expansion (PC16_PC26, *p* = 0.003; PC55_PC65, *p* = 0.002), nasal (RNW_LNW, *p* = 0.041) and pterygoid expansion (LLPt_RLPt, *p* = 0.004).

Finally, intergroup comparisons (Table 6) showed a statistically significant higher rate of expansion in the RME group for upper permanent molars (PC16_PC26, *p* = 00.025) but not for deciduous molars (PC55_PC65, *p* = 0.790) involved in the appliance. Moreover, RME showed statistically significant higher increases for nasal wall expansion (RNW_LNW, *p* = 0.004), while pterygoid expansion did not show significant differences (LLPt_RLPt, *p* = 0.849) in comparison between groups.

## 4. Discussion

Many past and actual studies used bidimensional imaging and dental casts to assess structural changes produced by RME or SME. Some authors studied the effects of maxillary expansion with appliances anchored to deciduous teeth [16,17,18], but to the best of our knowledge none of them compared RME with LE SME by CBCT analysis. Therefore, the present study has been carried out to tridimensionally compare the dental and skeletal effects produced by conventional RME and LE SME, showing differences between protocols. Based on the obtained results, i.e., that significant differences have been found on the dentoskeletal measurements, the null hypothesis was rejected.

Maxillary expansion is more efficient on dental than skeletal structures. This statement is very well documented in all studies carried out about RME and SME, as previously reviewed [19]. It seems that there are no significant differences between the increment of dental and skeletal widths in the subjects treated with RME or SME; even, RME guarantees greater expansion because of the use of longer expander screws compared with LE [20]. Furthermore, RME acts more orthopedically, being more effective in skeletal expansion compared with SME when evaluated by CBCT images [18]. In our study, considering nasal width changes, we observed the same results that were greater skeletal increase during rapid than slow expansion. When the ratio between skeletal and dental expansion is evaluated, the analysis of the scientific literature reported values of 36% and 35% [18] and 46% and 33% [21] for RME and SME, respectively; the ratios between intermolar and skeletal widths observed in the present study were only 20% for RME and 28% for SME. This inverted trend depends on whether skeletal expansion was quantified by pterygoid plates; both RME and SME showed the same expansion for this reference. However, studies performed on dental casts showed that SME with deciduous anchorage is effective in the correction of transversal dimension, increasing intermolar width as well as that of permanent molars not directly involved in the LE appliance, producing a self-orthodontic expansion [22]. In fact, if the same mathematical model was applied to the ratio between the intermolar width of first permanent molar and skeletal expansion, the results were 33% and 59% for RME and SME, respectively. Unfortunately, the differences between studies reveals different reference points and methods used to quantify the sutural opening. Essentially, orthopedic forces and molar tipping produced during RME emphasize the amount of dental expansion of anchoring teeth, whereas SME is effective in reducing partially the orthodontic effects. Despite rapid or slow protocols, skeletal expansion is reasonably stable in the long-term follow-up [23].

Our study provided an analysis of the short-term effectiveness of the two different techniques, but the long-term stability should be further investigated. The literature reports a relapse ranging from 0 to 45%, but the comparison between different investigations is complicated because of different study designs [24]. In general, it seems that maxillary expansion appears stable in the long-term follow-up independently from RME or SME. However, in addition to the stability, our short-term study needs to be compared with previous studies regarding dentoskeletal effects and potential side-effects of these two different approaches. Data from a recent meta-analysis suggested a greater intermolar expansion for RME compared with SME, as well as an increased buccal molar inclination and skeletal expansion [25]. Despite that, these results cannot be directly compared with ours, because none of the studies included in the meta-analysis performed the expansion with the device anchored to the deciduous teeth. However, the results are in accordance with the literature, in which the continuous light forces of SME were able to produce orthopedic changes comparable to those of the heavy forces of RME. Similar conclusions can be drafted regarding the side-effects produced by the two different analyzed protocols; although, it is not fully clear which one of the two maxillary expansion types leads to a greater commitment in these terms that would help the clinician to select the more appropriate treatment for alveolar bone health [26]. Once again, the main limitation to this analysis is the different anchorage used for maxillary expansion and the subsequent biases in the comparison with our results.

As previously reported, a recent systematic review and meta-analysis showed that RME produces a significantly greater expansion in posterior than anterior maxilla [25]; these results are in accordance with our findings, since incisor expansion was greater than expansion at the level of the nasal walls for RME compared with SME. Furthermore, our results regarding the reduction of buccal tipping of first permanent molars are confirmed by a similar study carried out with measurement obtained by dental models and postero-anterior cephalograms [12] that reported greater molar decompensation during LE SME than RME, even without statistical significance.

The biomechanics between LE and conventional appliances for RME should be considered in the technical explanation of dental and skeletal effects. An in vitro study regarding the mechanical properties of NiTi LE springs reported that forces exerted by the LE are different from the manufacturer’s ones [27]; this means that these forces may not necessarily open the suture but permit its remodeling. Nevertheless, the rate of SME is similar to the rate of bone formation at the level of the midpalatal suture, leading to a more physiological process compared with the disjunction of the suture provided by RME [28]. The accumulation of residual forces during RME therapy results in greater skeletal expansion, also because the high forces permit to prevail the frictional forces produced by the screw bending [2].

Palatal expansion on primary molars has several advantages for permanent molars, including spontaneous adjustment in their buccolingual inclination [29]. In the literature, the results on molar inclination are somewhat disparate, probably due to differences in samples, type of appliance and anchorage, amount of activation, and methodology. Deciduous anchorage avoids buccal tipping of the first permanent molars that is frequently observed when these teeth are used as an expander’s support, especially when RME is performed [16]. Buccal tipping is an undesirable effect shown when, during RME, the orthopedic forces cause the vertical pyramid-like opening of the maxillary halves, resulting in lateral alveolar tilting [30]. The clinical effect is the buccal movement of the crown of the anchorage teeth, even though this statement is not supported by an high level of evidence [25]. It seems that SME gives more time to upper molars to decompensate spontaneously with lower ones that are also indirectly affected by maxillary expansion both in short- and long-term periods [31]. In our study, even statistically and clinically non-relevant SME showed a greater palatal tipping variation, but it is unclear if this is due to a greater molar inclination before treatment or to the better capability of SME to determine a more gradual movement of permanent molars during the slow expansion on deciduous molars. This fact could explain the reason why SME seems more efficient in the ratio between dental and skeletal expansion, maybe because molars decompensate more and intermolar width results lesser due to a palatal tipping of the crowns. If this hypothesis is confirmed, SME on primary molars could find its indication in patients with severe buccal inclination of permanents molars, typical of maxillary hypoplasia in absence of posterior cross-bite, without the need for a preparatory appliance, such as a transpalatal bar, before starting expansion treatment.

Finally, the main limitation of this study was to not analyze independently the cross-bite and non-cross-bite sides. For this reason, more research and larger data are needed to increase our knowledge on long-term results and to better understand maxillary expansion treatment protocols and their skeletal and dental effects, especially about molar inclination control.

## 5. Conclusions

According to the results of the present study, the following conclusions might be drawn:

1. RME and SME are equally capable of producing both skeletal and dentoalveolar transverse expansion; deciduous teeth anchorage provides clinically significant transverse expansion causing less damage to permanent teeth;
2. RME produced more anterior expansion than SME, therefore it could be better suited for patients with respiratory dysfunctions and anterior dental crowding;
3. Molar inclination control is more superior for SME than RME, making SME recommended in patients with severe buccal inclination of upper permanent molars without the need for a preparatory appliance to molar decompensation;
4. SME provides a more controlled dental movement because of its constant release of forces, also eliminating the need for home activation.

## Figures and Tables

**Table 1:** Landmark definition.

**Skeletal Landmarks**		
Foramen spinosum (RFs, LFs)	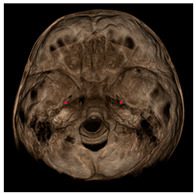	Geometric center of the upper and smallest circumference with defined borders viewed axially on the foramen spinosum.
ELSA	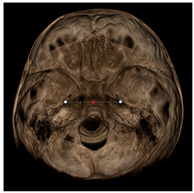	Midpoint on a line connecting left and right foramen spinosum.
Foramen ovale (RFo, LFo)	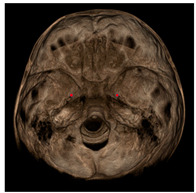	Most posterior, upper and lateral point on the posterior cortical bone with defined borders viewed axially on the foramen ovale.
Medial foramen magnum (MFM)	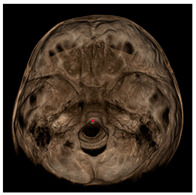	Most anterior point of the foramen magnum.
Lateral pterygoid plate (RLpt, LLpt)	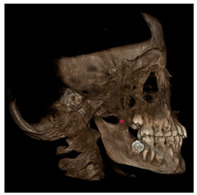	Most posterior border of the pterygoid lateral plate at the vertical level of the palatal shelves by using an axial slice showing as much of the palate surface as possible.
**Dental Landmarks**		
Pulp chamber (PC)	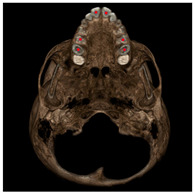	Center of the pulp chamber floor in upper first permanent molars (in all 3 planes of space). Center of pulp chamber floor in upper second deciduous molars (in all 3 planes of space). Tip of upper central incisor pulp chamber viewed sagittally.
Pulp horn (PH)	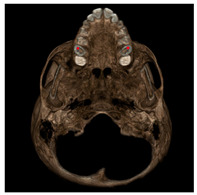	Tip of the mesio-buccal pulp horn of upper first permanent molars.
Furcation (FURCA)	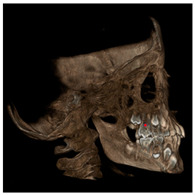	Furcation of upper first permanent molars.
**Nasal Landmarks**		
Nasal floor (RNF, LNF)	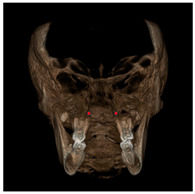	Junction of palatal cortical alveolar bone and cortical bone surrounding nasal cavity located in the coronal scan passing through PC16 point
Nasal wall (RNW, LNW)	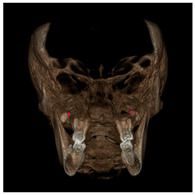	Most external point of the cortex bone separating the maxillary sinus and the nasal cavity located in the coronal scan passing through PC16 point

**Table 2:** Description of linear and angular measurements.

Measurement	Variable
PC16_PC26 (mm)	Permanent Molars Expansion
PC55_PC65 (mm)	Deciduous Molars Expansion
PC11_PC21 (mm)	Central Incisors Expansion
LLPt_RLPt (mm)	Lateral Pterygoid Expansion
RNW_LNW (mm)	Nasal Wall Expansion
RNF_LNF (mm)	Nasal Floor Expansion
FURCA16_PC16_PC26 (°)	Right Upper Molar Tip
FURCA26_PC26_PC16 (°)	Left Upper Molar Tip

**Table 3:** Intergroup comparison between Rapid Maxillary Expansion (RME) group and Slow Maxillary Expansion (SME) group at T1 (* *p* < 0.05).

Measurement	RME Group		SME Group		
	Mean	SD	Mean	SD	*p*
PC16_PC26 (mm)	40.9	3.7	41.1	2.5	0.621
PC55_PC65 (mm)	37.7	3.1	38.7	2.5	0.254
PC11_PC21 (mm)	7.9	0.6	9.0	3.5	0.518
LLPt_RLPt (mm)	50.5	4.2	49.5	28	0.649
RNW_LNW (mm)	28.9	2.3	28.1	2.0	0.197
RNF_LNF (mm)	17.3	5.2	16.7	3.1	0.447
FURCA16_PC16_PC26 (°)	116.9	25.6	135.8	9.4	0.019 *
FURCA26_PC26_PC16 (°)	112.7	31.1	136.2	9.6	0.043 *

**Table 4:** Rapid Maxillary Expansion (RME) intragroup comparison between T1–T2 (* *p* < 0.05).

Measurement	T1		T2		
	Mean	SD	Mean	SD	*p*
PC16_PC26 (mm)	40.9	3.7	44.8	3.4	0.001 *
PC55_PC65 (mm)	37.7	3.1	41.9	8.4	0.023 *
PC11_PC21 (mm)	7.9	0.6	8.7	1.0	0.001 *
LLPt_RLPt (mm)	50.5	4.3	51.8	4.0	0.006 *
RNW_LNW (mm)	29.0	2.3	31.2	2.4	0.001 *
RNF_LNF (mm)	17.3	5.5	19.0	6.0	0.157
FURCA16_PC16_PC26 (°)	116.9	25.6	116.3	32.4	0.647
FURCA26_PC26_PC16 (°)	112.7	31.2	113.5	40.6	0.913

**Table 5:** Slow Maxillary Expansion (SME) intragroup comparison between T1–T2 (* *p* < 0.05).

Measurement	T1		T2		
	Mean	SD	Mean	SD	*p*
PC16_PC26 (mm)	41.1	2.5	43.3	2.9	0.003 *
PC55_PC65 (mm)	38.7	2.5	43.2	2.6	0.002 *
PC11_PC21 (mm)	9.0	3.5	9.0	2.5	0.777
LLPt_RLPt (mm)	49.6	2.8	50.9	3.2	0.004 *
RNW_LNW (mm)	28.1	2.0	28.7	2.4	0.041 *
RNF_LNF (mm)	16.7	3.1	17.6	3.2	0.272
FURCA16_PC16_PC26 (°)	135.8	9.4	132.9	11.4	0.397
FURCA26_PC26_PC16 (°)	136.2	9.7	137.4	10.5	0.683

**Table 6:** Rapid Maxillary Expansion (RME) versus Slow Maxillary Expansion (SME) intergroup comparison of changes between T1–T2 (* *p* < 0.05).

Measurement	RME T2-T1		SME T2-T1		
	Mean	SD	Mean	SD	*p*
PC16_PC26 (mm)	3.9	1.9	2.2	1.7	0.025 *
PC55_PC65 (mm)	6.3	2.7	4.5	2.4	0.790
PC11_PC21 (mm)	0.8	0.7	0.0	1.4	0.058
LLPt_RLPt (mm)	1.3	1.8	1.3	1.3	0.849
RNW_LNW (mm)	2.2	1.6	0.6	1.7	0.004 *
RNF_LNF (mm)	1.7	4.8	0.9	2.3	0.184
FURCA16_PC16_PC26 (°)	−0.6	18.7	−2.9	11.9	0.323
FURCA26_PC26_PC16 (°)	0.8	25.8	1.3	8.4	0.879

## Data Availability

The data presented in this study are available on request from the corresponding author. The data are not publicly available due to privacy limitations.
